# The Martini Model in Materials Science

**DOI:** 10.1002/adma.202008635

**Published:** 2021-05-06

**Authors:** Riccardo Alessandri, Fabian Grünewald, Siewert J. Marrink

**Affiliations:** ^1^ Zernike Institute for Advanced Materials and Groningen Biomolecular Sciences and Biotechnology Institute University of Groningen Nijenborgh 4 Groningen 9747AG The Netherlands; ^2^ Present address: Pritzker School of Molecular Engineering University of Chicago Chicago IL 60637 USA

**Keywords:** coarse‐graining, Martini, molecular dynamics

## Abstract

The Martini model, a coarse‐grained force field initially developed with biomolecular simulations in mind, has found an increasing number of applications in the field of soft materials science. The model's underlying building block principle does not pose restrictions on its application beyond biomolecular systems. Here, the main applications to date of the Martini model in materials science are highlighted, and a perspective for the future developments in this field is given, particularly in light of recent developments such as the new version of the model, Martini 3.

## Graphical abstract

The Martini model, a coarse‐grained force field initially developed for biomolecular simulations, has found an increasing number of applications in materials science. The main applications of the Martini model in this field to date are highlighted, and a perspective for future developments is given, particularly in light of the new version of the model, Martini 3.

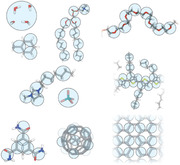

## 1 Introduction

Coarse‐grained (CG) force fields have gained a lot of popularity in the field of molecular dynamics (MD) simulations.^[^ ^1^, ^2^, ^3^ ^]^ By averaging out some of the atomistic degrees of freedom, they allow for a substantial alleviation of both the spatial and temporal limitations of all‐atom models. The Martini model^[^ ^4^, ^5^, ^6^ ^]^ is an example of a popular CG force field that has been incorporated by the worldwide user community to study a large variety of (bio)molecular processes.^[^ ^6^, ^7^ ^]^

In the Martini model, typically, four heavy atoms with their associated hydrogen atoms are grouped into one functional group, denoted as a CG bead. This effectively reduces the number of particles to be simulated in a system, increasing the simulation speed. In addition, the smoother CG energy landscape leads to faster overall dynamics and allows the use of larger time steps compared to all‐atom simulations. Together, this results in a significant increase in accessible length and time scales of a few orders of magnitude, albeit at somewhat reduced level of accuracy.

The CG beads represent small chemical fragments, and are used as building blocks for larger molecules. Parametrization of the nonbonded interactions between the CG beads is based on reproducing thermodynamic data such as free energies of transfer of organic compounds. In addition, reference all‐atom simulation data are used to derive effective bonded terms. Such a combination of top‐down and bottom‐up approaches enables the Martini model to distinguish different chemical species and form a useful bridge between atomistic and macroscopic scales.

From the first applications, purely concerned with lipids,^[^ ^8^, ^9^ ^]^ the Martini model has been applied to a vast amount of biomolecular systems. The compatibility with a wide library of existing molecules, which includes all major biomolecules such as proteins,^[^ ^10^ ^]^ sugars,^[^ ^11^ ^]^ DNA,^[^ ^12^ ^]^ or RNA,^[^ ^13^ ^]^ as well as an increasing amount of synthetic molecules,^[^ ^6^ ^]^ is one of the key strengths of the Martini model. It enables researchers to easily simulate complex many‐component systems and focus on more advanced simulation methodologies.

**Figure 1:**
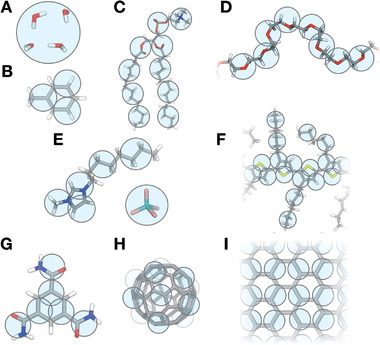
Martini mapping examples of selected molecules. A) Standard water particle representing four water molecules; B) the organic solvent toluene; C) dimyristoylphosphatidylcholine (DMPC) lipid; D) poly(ethylene oxide) (PEO); E) the 1‐octyl‐3‐methylimidazolium tetrafluoroborate [C_8_mim]^+^[BF_4_]^−^ ionic liquid; F) poly(3‐hexylthiophene) (P3HT); G) the 1,3,5‐benzenetricarboxamide (BTA) self‐assembling molecule; H) C_60_ fullerene; I) the surface of graphene. Martini CG beads are shown as cyan transparent beads overlaying the atomistic structure.

In recent years, the Martini model has found more and more applications in the field of materials science. In light of the underlying building block principle of the Martini model, there is no reason to restrict its applications to biomolecular systems. Indeed already more than a decade ago the Martini model has been applied to simulate polymeric systems.^[^ ^14^ ^]^ In principle, any molecule can be represented by Martini CG beads, as illustrated in **Figure** **1**. Based on this versatility, the Martini model is nowadays used to simulate a wide range of materials, including (block co)polymers, nanoparticle–polymer composites, organic electronic materials, ion‐conducting materials, self‐assembled supramolecular materials, ionic liquids, and others.

The use of CG models in material science is of course not new. In fact, some of the very first applications of CG models, such as the freely jointed chain models of Binder and co‐workers,^[^ ^15^ ^]^ already targeted polymer dynamics. Since those early days, a large plethora of CG models have been developed to model an equally large variety of materials.^[^ ^16^, ^17^, ^18^, ^19^, ^20^, ^21^, ^22^, ^23^ ^]^ Two major assets of the Martini model, that set it apart from most other CG models, are: i) the retaining of near atomic resolution, as opposed to more generic models that are frequently used to capture global system properties but are unable to offer a direct connection to chemical specificity, and ii) the broad range of compatible parameters available for different classes of molecules, enabling simulations of the ever expanding group of complex and hybrid materials as well as the interaction of materials with biological systems.

In the following sections, after summarizing Martini parametrization strategies tailored to material systems, we discuss the main application areas to date of the Martini model in materials science, illustrated with selected examples. While there are numerous Martini applications which involve the interaction of synthetic materials with biomolecules, we will not cover those here. The interested reader is referred to recent reviews and references therein.^[^ ^6^, ^24^, ^25^, ^26^ ^]^ We conclude with a perspective for the future developments in this field, in particular in light of the new version of the model, Martini 3, as well as recently developed tools to generate starting structures for polymeric systems and to allow constant pH simulations.

## 2 Parametrization Strategies

### 2.1 General Guidelines

Martini typically gathers groups of four non‐hydrogen atoms in CG beads—see Figure 1 for some representative mappings. The interactions between beads—described by a 12–6 Lennard‐Jones (LJ) potential—represent the nature of the underlying chemical groups and have been systematically parametrized to reproduce free energies of transfer of solutes between polar and nonpolar solvents. This parametrization target, the hallmark of the Martini force field, has been originally chosen because biomolecular processes such as lipid self‐assembly, protein–protein recognition, or membrane–peptide binding depend critically on the degree of hydrophobicity/hydrophilicity of the participating molecules. However, naturally the same critical dependence is found in the self‐assembly of synthetic molecules, polymer–solvent interactions, or more generally in soft matter systems.

There are four main particle types: polar (P), intermediately polar (N), apolar (C), and charged (Q). These types are in turn divided in subtypes based on their hydrogen‐bonding capabilities (with a letter denoting: d = donor, a = acceptor, da = both, 0 = none) or their degree of polarity (with a number from 1 = low polarity to 5 = high polarity). This gives a total of 18 particle types: the Martini building blocks. Such a building‐block approach—a discrete number of particles which interact using a limited number of interaction levels—provides compatibility of different Martini models and facilitates parametrization of new molecules, albeit limiting the quantitative accuracy of the force field. In addition to the regular Martini beads, smaller bead sizes (small and tiny beads) are used for groups that are represented at higher resolution such as ring‐like fragments.^[^ ^4^, ^6^ ^]^

In general, a Martini model for an arbitrary molecule can be generated as follows:

1) The atomistic structure is partitioned into a number of beads, maximizing the four‐to‐one mapping while preserving the symmetry of the molecule; smaller beads may be used to better represent the geometry of small ring‐like fragments.
2) Bead types describing the nonbonded interactions of the models are determined by comparing to already existing fragments or by computing free energies of transfer and selecting the best matching bead.
3) Bonded interactions, defined by a standard set of potential energy functions typical of classical force fields, are parametrized by comparing to atomistic simulations or experimental data.

The basic assumption underlying the Martini approach is that the thoroughly parametrized properties of the individual Martini bead types are transferable to the molecule as a whole when linked together to reproduce the overall topology of the desired molecule. This basic assumption entails some pitfalls,^[^ ^27^ ^]^ and hence requires validation, which commonly comes from either comparison to higher‐resolution atomistic simulations or to experimental data.^[^ ^6^ ^]^

Below we describe strategies based on the outlined general procedure but which have been found helpful for specific classes of molecules relevant to material systems such as polymers, nanoparticles, and surfaces. These may include specific reference data coming from atomistic simulations or experimental measurements to validate Martini models in this area.

### 2.2 Polymers

Martini polymers are applied in a wide range of studies on biomolecular and materials science systems. As first suggested by Rossi et al., parameters for Martini polymers are ideally generated by matching: (1) the free energy of transfer of the monomeric repeat unit; (2) bond and angle distributions of atomistic reference simulations; and (3) long‐range structural properties.^[^ ^28^ ^]^ Overall many carefully parametrized Martini polymers, such as polystyrene (PS),^[^ ^29^ ^]^ PEO,^[^ ^30^ ^]^ polyethylene (PE),^[^ ^31^ ^]^ and polypropylene (PP),^[^ ^31^ ^]^ have been validated by showing that they are able to reproduce a number of single‐chain properties. For example, the PS, PEO, as well as PEO–PPO models reproduce structural properties such as radii of gyration in different solvents.^[^ ^29^, ^30^, ^32^ ^]^ Indeed, validation of these properties in multiple solvents—so as to probe good, bad, and theta solvent conditions—is desirable. Furthermore, persistence lengths, structure factors, and polymer melt density constitute other properties which may be used as validation targets.

An important aspect to keep in mind when parametrizing (Martini) CG polymer models is that the use of torsion angle potentials borrowed from atomistic MD simulations is often unsuitable.^[^ ^33^ ^]^ Given the softer nature of angle potentials in CG models, conformations which lead to numerical instabilities can be encountered much more often, leading to impractical simulations. Bulacu and co‐workers have devised strategies, like the use of special bonded potentials such as the restricted bending potential (ReB)—implemented in GROMACS^[^ ^34^ ^]^—or virtual site‐aided definition of bonded terms, to combat this problem.^[^ ^33^ ^]^ These strategies allow such instabilities to be avoided and have been successfully applied to PEO,^[^ ^30^, ^33^ ^]^ PE,^[^ ^31^ ^]^ and other models. Using these strategies, simulations with chains of 500 repeat units over several tens of microseconds can easily be realized.^[^ ^30^ ^]^

The current library of Martini polymers comprises more than 50 different polymers. The models available range from simple linear polymers such as PEO (Figure 1D),^[^ ^14^, ^30^, ^35^, ^36^, ^37^ ^]^ nylon‐6,^[^ ^38^ ^]^ and PS,^[^ ^28^, ^29^, ^39^, ^40^, ^41^ ^]^ over branched and hyperbranched polymers such as (grafted) polyamidoamine (PAMAM)^[^ ^42^, ^43^, ^44^, ^45^, ^46^, ^47^, ^48^ ^]^ or polyethylenimine (PEI) dendrimers,^[^ ^49^, ^50^, ^51^, ^52^ ^]^ to conjugated polymers such as poly(3‐hexylthiophene) (P3HT, Figure 1F)^[^ ^53^ ^]^ and block copolymers^[^ ^32^, ^54^, ^55^ ^]^ such as PEO–PPO^[^ ^32^, ^56^ ^]^ or styrene– and diisobutylene–maleic acid copolymers.^[^ ^57^, ^58^ ^]^ Moreover, polymers have also been developed within the Dry Martini^[^ ^59^ ^]^ version of the force field: examples include PEO,^[^ ^60^ ^]^ PAMAM dendrimers,^[^ ^61^ ^]^ and charged polysaccharide.^[^ ^62^ ^]^ Within the Dry Martini model, water is represented implicitly rather than explicitly by CG particles. This is achieved by re‐parametrizing the nonbonded interactions between the Martini CG building blocks, such that the free energy of transfer between the implicit‐water and organic solvents is kept close to the original explicit‐water values. In addition, friction is introduced into the equations of motion by use of the GROMACS stochastic dynamics integrator.^[^ ^63^ ^]^ Through representing the waters implicitly Dry Martini can lead to a large speed‐up of simulations for which water is the main component.^[^ ^59^ ^]^ It has been shown to work well in the context of lipid bilayer simulations,^[^ ^59^ ^]^ and it has also been successfully used for simulating polymers.^[^ ^60^, ^61^, ^62^ ^]^ However, problems for example in polymer solvation have also been reported when using the Dry version of the model.^[^ ^64^ ^]^ In general, re‐balancing of the interactions and introduction of friction comes at a price. The new interactions are generally less accurate for polar beads and ions due to very weak interactions and the absence of strongly repulsive interactions.^[^ ^59^ ^]^ In addition speed‐up is limited by a slowed down diffusion as caused by the introduction of friction and limitations in parallelization due to domain decomposition in MD codes. Overall, for systems that are not largely composed of water, the speed‐up is typically around 2 or even less. In addition, in material science many environments are not composed of water, so they do not benefit from Dry Martini.

The fine degree of coarse‐graining of the Martini model means that there is no limitation to the topology of the polymer. Recently, a tool for easily generating topologies, as well as single chain and condensed phase starting structures of polymers has been developed by Grünewald and co‐workers—Polyply.^[^ ^65^, ^66^ ^]^ Polyply's internal library already contains several Martini polymer models from the literature but more models can be contributed via GitHub.^[^ ^65^ ^]^ This tool standardizes and greatly simplifies the generation and setup of Martini polymer systems, as discussed more extensively in the Outlook section.

### 2.3 Nanoparticles

Martini models for fullerene,^[^ ^67^, ^68^, ^69^ ^]^ carbon nanotubes (CNTs),^[^ ^70^, ^71^, ^72^, ^73^ ^]^ graphene^[^ ^74^, ^75^, ^76^ ^]^ or MXene^[^ ^77^ ^]^ flakes, clay nanoparticles,^[^ ^78^ ^]^ and functionalized nanoparticles^[^ ^79^, ^80^, ^81^ ^]^ have also been developed to study their interaction with both other synthetic or biomolecular systems. The C_60_ fullerene model developed by Monticelli represents a good parametrization strategy for nanoparticles.^[^ ^68^ ^]^ The model has been developed by matching experimental free energies of transfer between a wide range of solvents of different polarity and potentials of mean force (PMFs) of dimerization in water and octane.^[^ ^68^ ^]^ This thorough parametrization allowed the Martini fullerene model to reasonably reproduce solid‐state properties and lead to translocation PMF across a lipid membrane in good agreement with atomistic reference data. We note that the final model did not use a standard Martini CG bead but instead required some refinements. The thorough refinement of the parameters across a wide range of solvents, however, resulted in a model which could be used in other solvents, such as chlorobenzene, where a comparison to atomistic reference data showed excellent agreement,^[^ ^53^ ^]^ even though the C_60_ model had not been tested explicitly in chlorobenzene at the time of development. Hence, validation of nanoparticle models in different solvents, by means of comparison to experimental transfer free energies when available and PMFs of dimerization in different solvents is desirable when developing Martini models for these systems.

Of relevance for nanoparticle parametrization is also the work of Wang and Ferguson, who parametrized a Martini model for asphaltenes.^[^ ^82^ ^]^ They found the introduction of partial charges to represent the radial dipole moment of asphaltenes’ aromatic core to be critical to reproduce the T‐shaped stacking behavior observed in atomistic simulations.^[^ ^82^ ^]^

### 2.4 Surfaces

Simulations of Martini systems in materials science have also led to the development of models for graphite,^[^ ^83^, ^84^ ^]^ graphene,^[^ ^85^, ^86^ ^]^ and silica.^[^ ^64^ ^]^ As an example, the parametrization of the graphite model by Gobbo et al. used as reference experimental data enthalpies due to lack of experimental free energy data.^[^ ^84^ ^]^ Namely, the authors used: 1) enthalpies of adsorption of individual molecules from the gas phase on graphite, 2) wetting enthalpies of pure liquids, and 3) enthalpies of displacement of solutes (long‐chain organic molecules) from different solvents (heptane and phenyloctane) to graphite. The model required the development of a custom bead representing graphite with a 2‐to‐1 mapping scheme, which eventually could achieve semi‐quantitative reproduction of the experimental enthalpies.^[^ ^84^ ^]^

Besides the parametrization strategies and validation targets outlined above, several other application‐specific critical tests can be carried out to validate a particular Martini model. As we describe the applications in materials science to date in the following sections, we invite the reader to check the reference of interest to find out about further validation targets for the application of interest.

## 3 Example Applications

### 3.1 Polymeric Hydrogels

Polymeric hydrogels are networks composed of hydrophilic polymers that are covalently or physically cross‐linked. These polymer networks can swell taking up a multiple of their dry weight in water.^[^ ^87^ ^]^ They are frequently employed in drug delivery either as nanogel or macroscopic material.^[^ ^87^, ^88^ ^]^ Martini simulations were employed to understand: 1) interactions of hydrogels with the cargo molecules at molecular detail;^[^ ^89^ ^]^ 2) the gel response to environmental effects such as pH;^[^ ^90^ ^]^ 3) transport properties of the cargo inside a gel;^[^ ^91^ ^]^ and 4) effect of the salt concentration on the gel.^[^ ^92^, ^93^ ^]^

**Figure 2:**
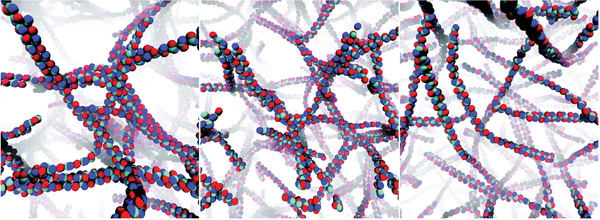
Typical conformations for a chitosan‐based hydrogel with a polymer concentration of 8.9%, and pH of >10.5 (left), 6.5 (middle), and <2.5 (right). Adapted with permission.^[^ ^90^ ^]^ Copyright 2017, Royal Society of Chemistry.

For example, Xu and Matysiak have developed a Martini model for chitosan and self‐assembled a chitosan hydrogel.^[^ ^90^ ^]^ Their findings indicate that physical cross‐linking patterns impact significantly the hydrogel's mechanical properties. In particular, increasing the polymer concentration or the pH translates into an increase of the elastic modulus of the system, as a consequence of changes in the cross‐linking patterns (**Figure** **2**). In another example, using a multi‐step protocol, protein imprinting of hydrogels was simulated using Martini. Protein imprinting proceeds by polymerizing monomers in the presence of a template protein to which monomers are reversibly coordinated. Following polymerization the gel is washed leaving—so the idea—specific coordination sides for the template protein. Subsequently, by swelling in solution the template protein or alike proteins adsorb more preferentially over random proteins.^[^ ^94^ ^]^ To mimic this process, Zadok and Srebnik first simulated coordination of acrylic Martini monomers with lysozyme and cytochrome c. Subsequently, using a reaction protocol within LAMMPS (Large‐scale Atomic/Molecular Massively Parallel Simulator), monomers were cross‐linked to form a gel. After removal of the unreacted monomers, a hybrid MD‐NVT grand‐canonical Monte Carlo simulation was used to swell the hydrogel by allowing the water content to change. Characterizing the interactions of both proteins with different hydrogels, they found that protein binding and selectivity is largely dependent on the nature of the polymer. However, it appeared lysozyme overall has the tendency of forming stronger interactions.^[^ ^89^ ^]^

### 3.2 Polymer Coatings and Glues

**Figure 3:**
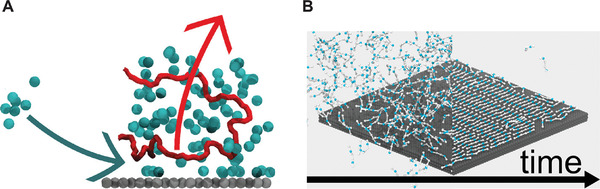
Adsorption of polymers and small molecules on surfaces. A) Adsorption of solvated polymer chains on a silica surface. Polymer solvation was found to play a key role for the adsorption of polymers on the silica substrate, highlighting the importance of an explicit description of the solvent in such studies.^[^ ^64^ ^]^ B) Self‐assembly on graphite: formation of long‐range ordered lamellar structures of self‐assembling (functionalized) alkanes physisorbed on graphite.^[^ ^100^ ^]^ A) Reproduced with permission.^[^ ^64^ ^]^ Copyright 2018, American Chemical Society. B) Reproduced with permission.^[^ ^100^ ^]^ Copyright 2019, American Chemical Society.

Another area where Martini polymers have been used extensively is to study polymer behavior at surfaces and interfaces. Studies have targeted for example polymer conformations at oil/organic solvent–water interfaces,^[^ ^95^, ^96^, ^97^ ^]^ surface water interfaces,^[^ ^64^, ^98^ ^]^ or even water/air interfaces.^[^ ^32^, ^55^ ^]^ Polymer behavior at interfaces is interesting in many applications among others for coatings or glues.^[^ ^64^, ^98^, ^99^ ^]^ For example, Perrin et al. used Martini to study conformations of poly(diallyldimethylammonium) (PDMA) and poly(acrylamide) (PAAm) absorbed to silica surfaces. The aim of the study was to investigate why PDMA glues to silica whereas PAAm does not regardless of their very similar chemical structure. According to their findings, PAAm is better solvated and therefore does not adhere to silica. They further found that dynamical properties of the polymer close to the surface can only be described by an explicit solvent model (**Figure** **3**A).^[^ ^64^ ^]^

Other studies have investigated the self‐assembly and self‐organization of various long‐chain (functionalized) alkanes on surfaces or at interfaces.^[^ ^84^, ^101^, ^102^, ^103^, ^104^, ^105^, ^106^, ^107^ ^]^ The Martini simulations performed by Piskorz et al. provided a microscopic view on the adsorption and subsequent rearrangement of alkanes on the surface to form long‐range ordered lamellar structures (Figure 3B).^[^ ^100^ ^]^ The assembly of porphyrin nanorings on graphite has also been explored.^[^ ^108^ ^]^

### 3.3 Microphase‐Separated Polymers

**Figure 4:**
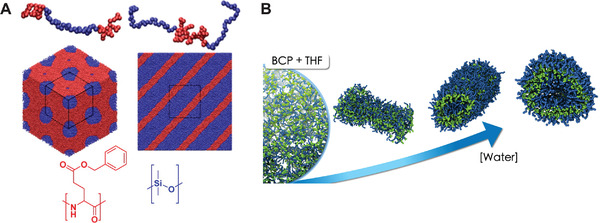
Block copolymer morphologies and self‐assembly. A) Hexagonally packed PDMS cylinders (left‐hand side) and lamellar morphology (right‐hand side). poly(y‐benzyl‐l‐glutamate) (PBLG) is rendered in red, and PDMS in blue.^[54]^ B) Morphology obtained from the self‐assembly of PEO‐*b*‐PBMA block copolymers (BCP) in water and THF mixtures. The morphology changes from dissolved chains or monomers in THF, over dispersed sheets or disk‐like aggregates, to vesicles as the fraction of water increases. The morphologies are also affected by the BCP concentration.^[^ ^123^ ^]^ A) Reproduced with permission.^[^ ^54^ ^]^ Copyright 2014, American Chemical Society. B) Reproduced with permission.^[^ ^123^ ^]^ Copyright 2020, Elsevier.

Although Martini reproduces well the phase behavior of small surfactants and polymers in aqueous solution,^[^ ^35^, ^109^, ^110^, ^111^, ^112^, ^113^, ^114^, ^115^, ^116^, ^117^ ^]^ predicting morphologies of large‐scale polymer—especially block copolymer—systems remains challenging. In particular microphase‐separated assemblies of copolymers are hugely important in many technological applications. For example, they are a key component for development of next‐generation batteries.^[^ ^118^ ^]^ Hence, simulations of microphase‐separated block copolymers have been performed with Martini^[^ ^54^, ^119^, ^120^, ^121^, ^122^ ^]^ In a first attempt, Johnson and co‐workers managed to self‐assemble in an unbiased fashion lamellar, micellar and cylindrical phases of a poly(dimethylsiloxane) (PDMS)–γ‐benzyl‐l‐glutamate block copolymer (**Figure** **4**A). Their simulations showed a strong correlation between the obtained morphology and the geometry and type of the side chain.^[^ ^54^ ^]^ In a similar study, Slimani et al. built a lamellar phase of a polyester gradient co‐block‐copolymer.^[^ ^120^ ^]^ These works demonstrate that studying these microphase‐separated polymer systems is in principle possible with Martini.

### 3.4 Block Copolymer Self‐Assembly

A large body of work has investigated the self‐assembly of different block copolymers in water or other solvents^[^ ^123^, ^124^, ^125^, ^126^, ^127^, ^128^, ^129^, ^130^, ^131^, ^132^, ^133^, ^134^, ^135^, ^136^ ^]^ Among these systems, the most studied with Martini are poloxamers^[^ ^32^, ^56^, ^137^, ^138^, ^139^, ^140^, ^141^, ^142^, ^143^, ^144^ ^]^ also known as pluronics, which are PEO–PPO–PEO amphiphilic triblock copolymers. Such polymers can form a wide range of aggregates ranging from bilayers, over micelles, to polymersomes, that is, synthetic vesicles, the latter ones being particularly studied for applications as nanocarrier devices for drug delivery. Related to the drug‐delivery applications but also of relevance for other technological uses, several Martini‐based works studied how small molecules (such as drugs or surfactants) self‐assemble with polymers or diffuse into polymer matrices.^[^ ^145^, ^146^, ^147^, ^148^, ^149^, ^150^, ^151^, ^152^, ^153^, ^154^ ^]^ In one study, Sharma and Dormidontova investigated the formation of polymer‐wrapped and polymer‐threaded worm‐like micelles as a function of polymer hydrophobicity and rigidity.^[^ ^151^ ^]^

In another study concerning block copolymer self‐assembly, Campos‐Villalobos et al. simulated PEO‐*b*‐poly(butylmethacrylate) (PBMA) copolymers, which are being studied for applications as nanostructured materials.^[^ ^123^ ^]^ Martini simulations of the self‐assembly of these block copolymers in water and tetrahydrofuran (THF) mixtures revealed the occurrence of a wide spectrum of mesophases (Figure 4B). The corresponding morphological phase diagram of this ternary system includes dispersed sheets or disk‐like aggregates, and spherical and rod‐like vesicles at low block copolymer concentrations, and bicontinuous and lamellar phases at high concentrations. Moreover, the THF/water relative content is found to play a crucial role on the self‐assembly kinetics and resulting morphologies.^[^ ^123^ ^]^

### 3.5 Nanoparticles

Martini parameters are available for a wide variety of nanoparticles including fullerenes,^[^ ^67^, ^68^, ^69^ ^]^ carbon nanotubes,^[^ ^70^, ^71^, ^72^, ^73^ ^]^ and gold^[^ ^79^, ^80^, ^155^ ^]^ among others. Apart from their importance to modeling organic electronics, which are the subject of the next section, they have been used to simulate polymer nanoparticle composites,^[^ ^78^, ^80^, ^95^, ^96^, ^155^, ^156^, ^157^, ^158^, ^159^, ^160^, ^161^, ^162^, ^163^, ^164^, ^165^ ^]^ self‐assembly of nanoparticles,^[^ ^79^, ^166^, ^167^, ^168^ ^]^ and solution processing of nanoparticles.^[^ ^76^, ^77^, ^85^, ^86^ ^]^

**Figure 5:**
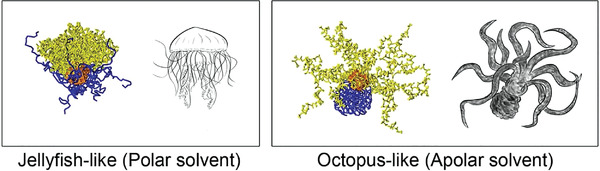
Nanoparticle–polymer systems. Jellyfish‐like and octopus‐like morphology of gold nanoparticles grafted with polymers in a polar and apolar solvent, respectively. Reproduced with permission.^[^ ^80^ ^]^ Copyright 2013, Wiley‐VCH.

For example, the behavior of poly(ethylene glycol) (PEG) grafted covalently or physically onto carbon nanotubes has been studied in some detail.^[^ ^157^, ^158^, ^159^, ^161^ ^]^ In addition to carbon‐based nanoparticles, inorganic nanoparticles with grafted polymers are an interesting nanomaterial with potential applications in sensoring, microfluidics, and smart surfaces, to name some examples.^[^ ^80^ ^]^ They are especially interesting for their response to different solvents. Within the Martini framework, in particular gold nanoparticles have received a lot of attention.^[^ ^80^, ^95^, ^96^, ^162^, ^164^, ^169^, ^170^ ^]^ For example, Dong and Zhou have studied the solvent behavior of differently composed PEO‐*b*‐PS block copolymers attached to gold nanoparticles. They found varying the composition of the block copolymer leads to a variety of different morphologies. Some of them were the expected sphere–shell like conformations where the polymers extend or collapse in a trivial fashion onto the nanoparticles. On the other hand, Dong and Zhou also identified some nontrivial conformations described as rings, buckles, and sectorially arranged chains (**Figure** **5**).^[^ ^80^ ^]^ Dahal and co‐workers have studied in detail PEO‐grafted gold nanoparticles, investigating hydration and structural properties as a function of PEO chain length and grafting density,^[^ ^162^, ^164^ ^]^ with observed properties in agreement with experimental data but providing a microscopic view on such nanoparticle–polymer composites. These examples highlight the possibility of using Martini to scan many different compositions for such systems and optimize a target behavior without the need for experimentally synthesizing all of the structures.

The study of materials composed of self‐assembled coated nanoparticles is another interesting effort. Solids formed by such nanoparticles form a versatile class of hybrid materials in which both the nanoparticle core and the organic ligand shell can be tuned, leading to a variety of materials. For example, Martini as been used to simulate single‐layer coated nanoparticle membranes,^[^ ^166^ ^]^ or nanoparticle superlattices for energy applications.^[^ ^168^ ^]^ Additionally, the dispersion of CNTs and 2D materials, such as graphene and MXene, in surfactant aqueous solutions^[^ ^76^, ^77^, ^85^, ^86^ ^]^ has been investigated. Understanding and optimizing such dispersion is of paramount importance for the processing of these materials; in the case of the 2D material MXene, Li et al. probed three typical surfactants with different structural characteristics, finding that the surfactant with long hydrocarbon chain and positively charged head group can form stable bilayers at the surface with MXene, which has implications for the thermal energy dissipation of the 2D material.^[^ ^77^ ^]^ In a similar vein, the surfactant‐templated formation of porous silica materials has been investigated in detail by Jorge, Pérez‐Sánchez, Gomes, and co‐workers.^[^ ^171^, ^172^, ^173^, ^174^, ^175^ ^]^ For example, their studies highlighted the critical role of silica oligomers in bridging micelles and hence allow the formation of aggregates.^[^ ^172^ ^]^ Additionally, the self‐assembly of asphaltenes^[^ ^82^, ^176^, ^177^, ^178^, ^179^ ^]^ has also been investigated, as their stability in solution strongly depends on temperature, pressure, and composition and is important in process and energy engineering. Other applications in the field of nanoparticle composite materials include polymer/graphene,^[^ ^75^ ^]^ polymer/graphite,^[^ ^180^ ^]^ polymer/clay^[^ ^78^ ^]^ composites, and polymer–CNT–protein matrices for applications in the field of tissue regeneration.^[^ ^181^ ^]^

### 3.6 Organic Electronics

The morphology of the organic material that constitutes the active layer of organic electronic devices is a critical parameter for the functioning of such devices. Computational modeling of the morphology represents a fundamental step toward an increased rational approach to the design of high‐performance organic materials for electronic applications.^[^ ^182^, ^183^ ^]^ It is possible to model the morphology of organic electronic materials with Martini, in particular to obtain and characterize morphologies, which are often composed of more than one organic semiconductor;^[^ ^53^, ^184^, ^185^, ^186^, ^187^, ^188^, ^189^, ^190^ ^]^ and to subsequently backmap^[^ ^191^ ^]^ the obtained CG morphologies to atomistic resolution, a step often useful in order to perform fine‐grained calculations aimed at evaluating the electronic properties of such materials.^[^ ^53^, ^184^, ^192^, ^193^, ^194^ ^]^ Martini models have been already developed for many prototypical organic semiconductors used in organic electronic devices, such as conjugated polymers,^[^ ^53^, ^114^, ^195^ ^]^ small conjugated molecules,^[^ ^184^, ^185^, ^196^, ^197^ ^]^ and C_60_ fullerene^[^ ^67^, ^68^ ^]^ and some of its derivatives.^[^ ^53^, ^185^, ^193^ ^]^ Arguably one of the most popular subfields of organic electronics is organic photovoltaics. Systems such as P3HT:DiPBI,^[^ ^184^ ^]^ P3HT:PCBM,^[^ ^53^, ^186^, ^187^, ^188^, ^189^, ^198^ ^]^ PBDB‐T:F‐ITIC,^[^ ^190^ ^]^ and P3HT:PTEG‐1^[^ ^193^ ^]^ have already been simulated with Martini (DiPBI is diperylene bisimide, PCBM is phenyl‐C61‐butyric acid methyl ester, PTEG‐1 is triethyleneglycol‐2‐phenyl‐*N*‐methyl‐pyrrolidino[[3′,4′:1,2]][C60]fullerene, PBDB‐T is poly[(2,6‐(4,8‐bis(5‐(2‐ethylhexyl)thio‐phen‐2‐yl)‐benzo[1,2‐b:4,5‐b0]dithiophene))‐*alt*‐(5,5‐(10,30‐di‐2‐thienyl‐50,70‐bis(2‐ethylhexyl)benzo[10,20‐c:40,50‐c0]dithiophene‐4,8‐dione))], and F‐ITIC is ITIC‐F = fluorinated (2,2′‐[[6,6,12,12‐tetrakis(4‐hexylphenyl)‐6,12‐dihydrodithieno[2,3‐d:2′,3′‐d′]‐s‐indaceno[1,2‐b:5,6‐b′]dithiophene‐2,8‐diyl]‐bis‐[methylidyne(3‐oxo‐1H‐indene‐2,1(3H)‐diylidene)]]bis‐[propanedinitrile])). Simulations of neat P3HT^[^ ^192^ ^]^ have also been performed, while more organic semiconductor mixtures have been tested in the context of organic thermoelectric devices^[^ ^185^, ^196^ ^]^ and organic mixed ion–electron conductors (which will be described as part of the next section).^[^ ^194^, ^195^, ^199^, ^200^, ^201^ ^]^

**Figure 6:**
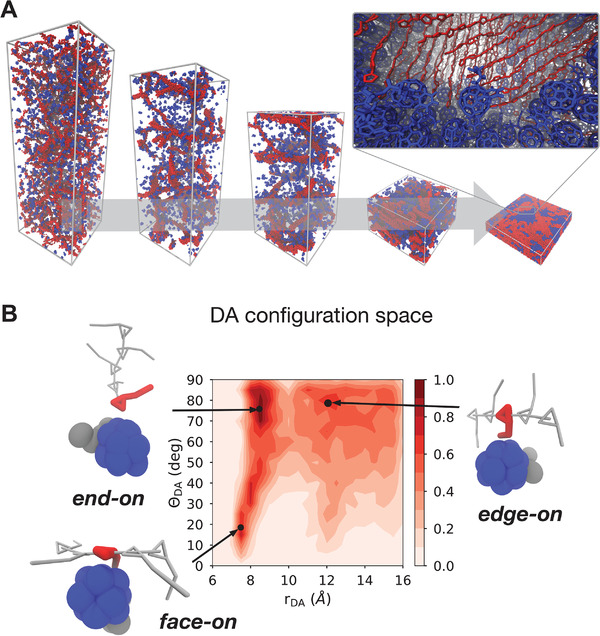
Simulation of organic electronic materials. A) Bulk heterojunction morphologies from solvent evaporation simulations for a P3HT–PCBM mixture.^[^ ^53^ ^]^ The inset shows the resulting atomistic structure obtained via backmapping. P3HT polymer chains are rendered in red and PCBM molecules in blue, respectively. B) Molecular orientations at the donor–acceptor (DA) interface can be resolved, also as a function of molecular features and processing conditions.^[^ ^193^ ^]^ A) Reproduced with permission.^[^ ^53^ ^]^ Copyright 2017, American Chemical Society. B) Adapted with permission.^[^ ^193^ ^]^ Copyright 2020, Wiley‐VCH.

When modeling the morphology of organic electronic materials, simulating fabrication processes, such as solution‐processing and thermal annealing, is an important step to be taken into account and which can be studied to obtain in silico insights. The solvent evaporation process which takes place during the fabrication of organic thin films can be simulated by simulating “bulk” evaporation, as first shown by Lee and Pao using a supra CG model.^[^ ^202^ ^]^ Similar solvent evaporation simulations have been applied to simulate the prototypical polymer:fullerene photovoltaic blend—P3HT:PCBM—at the Martini level by Alessandri et al. (**Figure** **6**).^[^ ^53^ ^]^ Other Martini organic semiconductor thin films have been solution‐processed in silico.^[^ ^186^, ^187^, ^188^, ^189^, ^193^, ^194^, ^195^, ^198^ ^]^ In particular, Alessandri et al. studied the evolution of the morphology of P3HT:PCBM blends as a function of the molecular weight of P3HT, the solvent evaporation rate, and thermal annealing. In agreement with experiments, thermal annealing and slower evaporation rates lead to larger phase separation and increased crystallinity of the P3HT phase. The crystallinity of P3HT could be probed by computing scattering signals, which were found to be in qualitative agreement with experimental data.^[^ ^53^ ^]^ The too‐large size of S‐beads, however, prevents a quantitative reproduction of the stacking distance between the polythiophene backbones.^[^ ^53^ ^]^ Besides allowing quantification of the degree of crystallinity, computing X‐ray scattering signals of simulated morphologies allows for comparison to experimental data. Other works made comparison between CG and X‐ray scattering data,^[^ ^53^, ^187^, ^194^, ^203^ ^]^ or to scanning electron microscopy^[^ ^53^ ^]^ or atomic force microscopy (AFM)^[^ ^185^, ^203^ ^]^ images. Martini simulations allow a range of parameters that are known to affect the morphology of organic thin films in the lab, such as the weight ratio of the components,^[^ ^187^ ^]^ polymer polydispersity,^[^ ^188^ ^]^ molecular weight,^[^ ^53^, ^187^ ^]^ and post‐evaporation^[^ ^53^, ^187^ ^]^ and pre‐evaporation^[^ ^187^ ^]^ heating treatments, to be scanned. Moreover, once morphologies have been generated, their macroscopic properties, such as mechanical properties,^[^ ^189^ ^]^ or microscopic features, such as the molecular orientations at the interfaces between the two blended organic semiconductors^[^ ^193^ ^]^ (Figure 6B), can be investigated, possibly as a function of the above parameters. Finally, other works have looked at the solubility of small molecules used as dopants in environments of different polarity,^[^ ^196^ ^]^ another application that is suited to the Martini model.

An important advantage of obtaining morphologies at the Martini level is the possibility of directly backmapping^[^ ^191^ ^]^ the CG morphologies to atomistic resolution (Figure 6A, inset), hence obtaining atom‐resolved structures which take into account the self‐organization process which occurs during the processing of an organic blend.^[^ ^53^ ^]^ Indeed, large‐scale morphologies have been backmapped to atomistic resolution in order to compute, by means of (semi‐empirical) quantum chemical calculations: UV–vis spectra,^[^ ^184^, ^192^ ^]^ energy levels taking into account the local molecular environment,^[^ ^193^ ^]^ and charge carrier hopping rates^[^ ^194^ ^]^ for charge transport calculations.

### 3.7 Ion‐Conducting Organic Materials

**Figure 7:**
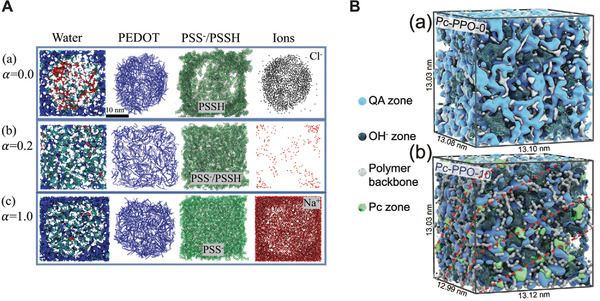
Simulation of morphologies of ion‐conducting materials. A) Representative morphologies of poly(3,4‐ethylenedioxythiophene):polystyrene sulfonate (PEDOT:PSS) systems as a function of the (PSS) deprotonation level, α, which is a function of the pH.^[^ ^199^ ^]^ For α = 0 all PSS chains are protonated (PSSH), vice versa for α = 1. The water phase (left‐hand side) is colored in red, blue, or cyan, when the water molecules are within a distance of 6 Å  from PEDOT, PSS, or both, respectively. B) Martini models of anion exchange membranes for fuel cells.^[^ ^210^ ^]^ Introduction of 10% of quaternary ammonium (QA) phthalocyanine (Pc) groups (Pc‐PPO‐10) (a) induces a more structured self‐assembly than the random morphology formed by Pc‐PPO‐0 (where QA‐Pc groups are not present) (b), leading to enhanced hydroxide (OH^−^) conductivity. Here, PPO stands for poly(2,6‐dimethyl‐1,4‐phenylene oxide). A) Adapted with permission.^[^ ^199^ ^]^ Copyright 2019, Royal Society of Chemistry. B) Adapted with permission.^[^ ^210^ ^]^ Copyright 2020, Wiley‐VCH.

Martini has also been used to investigate^[^ ^194^, ^195^, ^199^, ^200^, ^201^, ^204^ ^]^ organic mixed ion–electron conductors, which are soft (semi‐)conductors—often polymers—that readily solvate and transport ionic species.^[^ ^205^, ^206^ ^]^ Applications of such systems include organic electrochemical transistors for biological interfacing and neuromorphic devices, among others.^[^ ^207^ ^]^ Martini allows the chemical specificity to be retained when describing such materials, in contrast to more generic bead–spring CG models.^[^ ^208^ ^]^ A Martini model exists for the workhorse system of this field: poly(3,4‐ethylenedioxythiophene):polystyrene sulfonate (PEDOT:PSS).^[^ ^199^ ^]^ Modarresi, Zozoulenko, and co‐workers spearheaded Martini‐based studies in this field by developing a model for PEDOT and investigating ion diffusion in morphologies of PEDOT:Tos, a system made of PEDOT chains and tosylate (Tos), a negatively charged molecular counterion.^[^ ^195^ ^]^ The same authors, combining PEDOT with the available^[^ ^209^ ^]^ PSS model, went on to investigate PEDOT:PSS morphologies in detail.^[^ ^199^, ^201^ ^]^ The simulations allowed the effect of pH on the morphology of in silico solution‐processed PEDOT:PSS thin films to be studied. Changes in pH were found to greatly affect the morphology (**Figure** **7**A), and in turn the distribution of the 5–15 weight % of water content in the polymer film, which is of critical importance for ion diffusion. Once again, the possibility offered by Martini of easily combining models allowed Mehandzhiyski and Zozoulenko to simulate PEDOT:PSS/cellulose^[^ ^211^ ^]^ composite paper;^[^ ^203^ ^]^ such paper can be used in applications such as fuel cells, sensors, and batteries, among others.^[^ ^212^ ^]^ The authors could pinpoint the most likely configuration of PEDOT and PSS/PSSH chains around cellulose by comparing the simulation results to AFM images.^[^ ^203^ ^]^ They could identify the most likely morphology observed in the experiments, namely a bead‐like structure caused by PEDOT aggregates on the fibril which are separated by regions with a lower density.^[^ ^203^ ^]^

The works presented above on mixed ion–electron conductors partly build on earlier developments of Martini models for polyelectrolytes, which include models for polystyrene sulfonate (PSS),^[^ ^209^, ^213^ ^]^ poly(diallyldimethylammonium) (PDADMA),^[^ ^209^ ^]^ and more recently, partially hydrolyzed polyacrylamide (HPAM).^[^ ^214^ ^]^ In the case of such highly charged systems, polarizability introduced for water and other beads has been shown to be important.^[^ ^215^, ^216^, ^217^, ^218^, ^219^ ^]^ Hence, when charged interactions are expected to be important, it is recommended that models are developed in the context of the polarizable^[^ ^215^, ^216^ ^]^ water model. For example, Vögele et al. have shown that the Martini model of PSS used in polarizable water is able to accurately describe ion distributions around the polymer and reduction in dielectric screening. These important properties are not reproduced in regular Martini water.^[^ ^209^ ^]^

Furthermore, Martini has been applied to model ion‐conducting polymeric materials used as ion‐exchange membranes for fuel cell applications.^[^ ^210^, ^220^, ^221^, ^222^, ^223^, ^224^, ^225^, ^226^ ^]^ Proton‐exchange‐membrane fuel cells use acid polyelectrolytes, such as Nafion, as the membrane material. The membranes are formed by solution‐processing techniques. In this context, Mabuchi and co‐workers investigated dilute solutions of Nafion ionomers, the initial stage of solution processing: they studied ionomer self‐assembly in mixtures of 1‐propanol and water and probed the effects of ionomer concentration, alcohol content, and inclusion of salt.^[^ ^223^, ^225^ ^]^ Goncalves et al. studied instead how cavities nucleate and grow in a hydrated Nafion membrane subject to mechanical deformation, obtaining a nanoscale view on the mechanical properties of such membranes.^[^ ^221^ ^]^ Next to proton‐exchange membranes, also alkaline anion‐exchange membranes, which transport instead alkaline anions (usually hydroxide), have been modeled with Martini.^[^ ^210^, ^222^, ^224^ ^]^ Pan et al., for instance, screened for different structural designs of polymer electrolytes which would increase hydroxide mobility, a key performance parameter. The prediction was implemented experimentally and found to lead to increased hydroxide mobility, which reached efficiencies as high as the proton mobility in the more developed Nafion‐based proton exchange membranes.^[^ ^222^ ^]^ Finally, recently Yang and co‐workers used the Martini model to microscopically investigate the impact of adding quaternary ammonium phthalocyanine (Pc) groups into anion exchange membranes based on poly(2,6‐dimethyl‐1,4‐phenylene oxide) for alkaline fuel cells.^[^ ^210^ ^]^ The self‐assembly of Pc helps structuring the anion exchange membrane, increasing its hydroxide conductivity (Figure 7B).^[^ ^210^ ^]^

### 3.8 Self‐Assembled Supramolecular Materials

Self‐assembly of molecular building blocks into supramolecular materials holds much promise for a range of potential applications in nanotechnology.^[^ ^227^, ^228^ ^]^ Molecular building blocks that are very popular are short peptides (2–10 residues) and peptide conjugates, which can give rise to a large variety of biocompatible nanostructures.^[^ ^229^ ^]^ Many groups have explored their self‐assembly process by leveraging the Martini model.^[^ ^230^, ^231^, ^232^, ^233^, ^234^, ^235^, ^236^, ^237^, ^238^, ^239^, ^240^, ^241^, ^242^, ^243^, ^244^, ^245^, ^246^, ^247^, ^248^, ^249^, ^250^, ^251^, ^252^ ^]^ Besides allowing simulation of the self‐assembly and growth, Martini is also particularly suited for high‐throughput applications. An example of such a high‐throughput application in the area of peptide‐based supramolecular materials is the work of Frederix et al. who simulated all 8000 combinations of tripeptides.^[^ ^239^ ^]^ The prediction of self‐assembling and non‐assembling peptide sequences coming from the Martini simulations was verified by a full experimental characterization, showing the predictive power of Martini in this area. The approach allowed the extraction of guidelines for new peptide materials.^[^ ^239^ ^]^ The modularity of the Martini model also allows peptides to be easily combined with other molecular moieties. Accordingly, Mansbach and Ferguson built models for π‐conjugated peptides, which are promising bioelectronic materials due to their optoelectronic properties, and studied their self‐assembly in detail.^[^ ^253^, ^254^, ^255^, ^256^ ^]^

**Figure 8:**
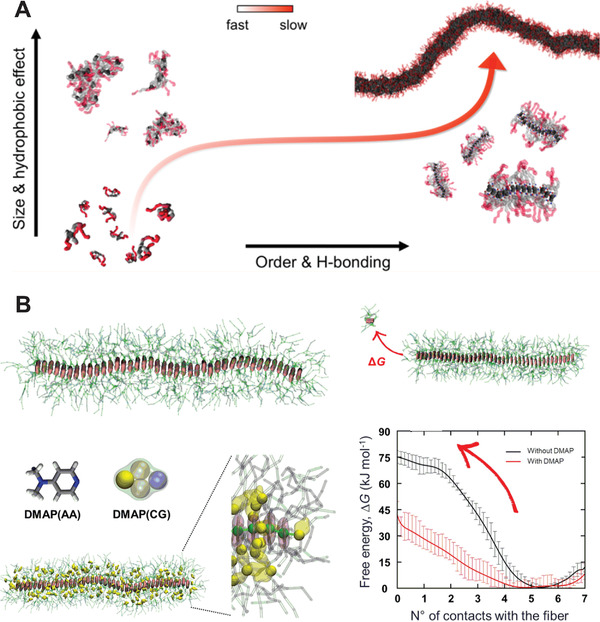
Examples of supramolecular polymers. A) Formation of BTA supramolecular polymers, as resolved by Martini CG simulations, proceeds via an initial fast aggregation followed by a slower reorganization and fiber growth.^[^ ^257^ ^]^ B) Modeling of porphyrin‐based supramolecular copolymers: the presence of DMAP small‐molecules eases porphyrin monomer exchange in and out the fiber, as quantified by the free energy profiles.^[^ ^264^ ^]^ A) Reproduced with permission.^[^ ^257^ ^]^ Copyright 2017, American Chemical Society. B) Adapted with permission.^[^ ^264^ ^]^ Copyright 2017, American Chemical Society.

Besides peptide‐based compounds, studies on other supramolecular systems have been reported in more recent years. An important example are 1,3,5‐benzenetricarboxamide (BTA)‐based supramolecular polymers, synthetic supramolecular materials which have been studied in detail by Bochicchio, Pavan, and co‐workers.^[^ ^257^, ^258^, ^259^, ^260^, ^261^, ^262^, ^263^ ^]^ Two Martini models for the BTA core were developed, both capturing the step‐wise cooperative polymerization mechanism which leads to the formation of supramolecular fibers (**Figure** **8**)^[^ ^257^ ^]^: BTA monomers initially aggregate quickly in water due to hydrophobic interactions; the disordered aggregates formed then reorganize into directional oligomers on a slower time scale; such ordered oligomers then fuse to form and elongate the supramolecular fiber on an even slower time scale. In the more refined model, additional charged particles were introduced to improve the stacking interactions between the BTA cores,^[^ ^257^ ^]^ similarly to the ones introduced within amino acid side chains by de Jong et al. in the polarizable version of the Martini protein model.^[^ ^219^ ^]^ The refined model allows for accurate monitoring of hydrogen‐bonding between the BTA monomers, while the simpler model—where such charges are omitted—is recommended for studies of interactions of BTA‐based assemblies with other (macro)molecules.^[^ ^257^ ^]^ A Dry Martini^[^ ^59^ ^]^ version of the BTA model has also been put forward by the same authors.^[^ ^259^ ^]^ There are several applications of structural variants of the BTA supramolecular polymer,^[^ ^258^, ^260^, ^261^, ^262^ ^]^ some in combination with enhanced sampling and machine‐learning techniques.^[^ ^258^, ^262^ ^]^ For example, Martini‐based well‐tempered metadynamics simulations were used to investigate monomer exchange in and out of the fibers, finding a central role of defects on the supramolecular structure in this process.^[^ ^258^ ^]^ Being able to characterize these defects may thus be important to control the dynamic behavior and properties of such systems: Gasparotto and co‐workers used machine‐learning techniques to systematically identify and compare such defects in this class of supramolecular materials.^[^ ^262^ ^]^

Other supramolecular polymer aggregates studied include: benzotrithiophene (BTT)‐based supramolecular fibers,^[^ ^265^ ^]^ azobenzene‐containing monomers, which assemble in a supramolecular tubule,^[^ ^266^ ^]^ and porphyrin‐based supramolecular polymers.^[^ ^264^ ^]^ In the latter study, Martini‐based well‐tempered metadynamics allowed Jung et al. to quantify the effect of a small molecule, DMAP, on the monomer exchange from the fiber (Figure 8B).^[^ ^264^ ^]^ The simulations showed that DMAP molecules interfere with the monomer‐monomer interactions at the fiber ends by first penetrating in between the monomer porphyrin cores and then facilitating monomer dissociation from the fiber end.^[^ ^264^ ^]^ Other supramolecular material systems that have been simulated with Martini include supramolecular block copolymers,^[^ ^267^, ^268^ ^]^ peptide‐based supramolecular polymers chemically linked to spiropyran‐based networks,^[^ ^269^ ^]^ poly‐catenanes,^[^ ^270^ ^]^ peptoid‐based nanomaterials,^[^ ^271^ ^]^ supramolecular macrocycle fibers,^[^ ^272^ ^]^ responsive conjugated polymers,^[^ ^273^ ^]^ platinum complexes,^[^ ^274^, ^275^ ^]^ supramolecular polymer hydrogels,^[^ ^276^ ^]^ and light‐harvesting double‐walled nanotubes.^[^ ^277^ ^]^

### 3.9 Green Solvents

Historically, the Martini model was developed for the simulation of biological membranes formed by lipids. With lipids being one special class of surfactants, early on Martini was extended to simulate the assembly and interaction of other synthetic ionic and nonionic surfactants.^[^ ^35^, ^83^, ^113^, ^278^, ^279^, ^280^, ^281^, ^282^, ^283^ ^]^ Recently, interest in surfactants has renewed as ionic liquids (ILs) have attracted much attention for their use as biocompatible and green solvents and co‐solvents. This has led several authors to use Martini to simulate the self‐assembly of IL mesophases,^[^ ^284^, ^285^ ^]^ the process of IL‐mediated extractions,^[^ ^285^, ^286^ ^]^ as well as to guide the design of de novo molecules.^[^ ^286^ ^]^

The use of ionic liquids in applications such as extractions is directly linked to the phase behavior of the ionic liquid as well as to the emerging phase behavior when combined with co‐solvents. Therefore, understanding and predicting these phases is an important step toward efficient computational solvent design. Martini simulations have very recently been used to unravel the phase behavior of pure ILs, ILs in water, and mixtures of ILs with other molecules.^[^ ^142^, ^284^, ^285^, ^287^ ^]^

For example, Pérez‐Sánchez and co‐workers used Martini simulations to understand the temperature‐dependent effects of adding surface active ILs to Pluronic block‐copolymer water mixtures. They were able to elucidate how micelle formation and aggregation changes with Pluronic block‐copolymer composition and type of IL. Their results were well in line with experimental cloud‐point measurements. This example demonstrates that Martini simulations can be used to study temperature dependence of ionic liquid phase behavior, at least at a qualitative level. Also other studies have successfully used Martini simulations to investigate temperature‐dependent effects in the context of ILs.^[^ ^284^, ^285^, ^288^ ^]^

**Figure 9:**
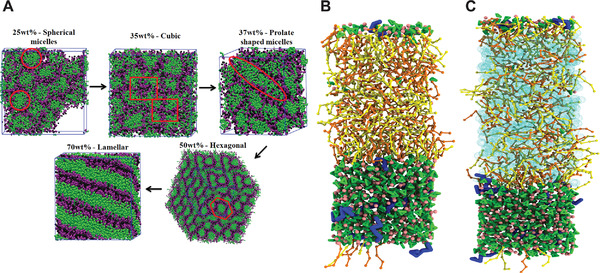
Martini modeling of ionic liquids. A) Mesophases formed by the ionic liquid ([C_10_mim]^+^[BF_4_]^−^) water mixtures simulated using Martini for ionic liquids. B,C) Snapshots of the final Martini 3 simulation box of the extraction of polyunsaturated fatty acids from fish oil with an ionic liquid.^[^ ^285^ ^]^ In the system (C), octane is added with respect to the composition of system (B), to test the stability of the biphasic system through the addition of co‐solvent. The color coding is as follows: the IL cation representing the imidazolium ring and the alkyl tail is in green, while the IL anion in pink. The polyunsaturated fatty acids to be extracted are in blue, while the palmitic and oleic acids forming the fish oil phase are in yellow and orange, respectively. Octane is depicted in cyan. A) Reproduced with permission.^[^ ^284^ ^]^ Copyright 2020, Elsevier. B) Adapted with permission.^[^ ^285^ ^]^ Copyright 2020, Royal Society of Chemistry.

As another example, Crespo et al. investigated the phase diagram of [C_*n*_mim]^+^[BF_4_]^−^ water mixtures. Their simulations show that this type of ILs displays a rich phase behavior as a function of the water content but also temperature in agreement with experimental data where available (**Figure** **9**A). The authors also compared the performance of Martini to bottom‐up derived CG models. They found that Martini outperforms those models when it comes to transferability from the neat state to mixtures with water. A similar conclusion was reached by Potter et al. who investigated the phase behavior of a type of a non‐ionic chromonic molecule. In their study, a direct comparison is made between a bottom‐up CG model following the approach of Lu and co‐workers^[^ ^289^ ^]^ and the top‐down Martini approach to CGing. Only with Martini they were able to simulate the complete phase diagram, whereas the structural CG model showed severe limitations at higher concentrations.^[^ ^283^ ^]^

Whereas the previous studies focused on ILs in co‐solvency with water, Vazquez‐Salazar et al. used the Martini 3 model to simulate pure [*C*_*n*_ *mim*[*CL*]]. They observed that Martini well reproduces the system density as function of alkyl chain length and temperature. In addition, the simulations showed the clear formation of dynamic local organization of the IL, so‐called nanodomains, which are an important feature of this type of ILs. Furthermore, the authors demonstrated that the Martini model is able to capture a phase transition of [*C*_12_ *mim*[*CL*]].^[^ ^285^ ^]^ This phase transition has also been determined experimentally and takes place at around 324.75 K. The CG model produced a clear phase transition at 325 K, in excellent agreement with experiment.

While the phase behavior of ILs and their mixtures is an important feature for extractions and applications, it is only indirect evidence for extraction efficiency of a particular IL. Vazquez‐Salazar et al. also demonstrated that it is feasible to simulate the extraction process directly by creating a biphasic system of the IL and the solvent phase from which the solute is to be extracted (Figure 9B,C). Initially all solute molecules are in the solvent phase, but after 6 μs of simulation the solutes distribute between the two phases. From the analysis of the solute and solvent density profiles, extraction efficiency and selectivity could be computed. Using this protocol, extraction of benzene and polyunsaturated fatty acids from model oil phases was characterized. It was found that the simulations, based on the new Martini 3 version, are well in line with the trends observed in experiment.

In a different study, Huet and co‐workers designed de novo ILs, so called zwitterionic liquids, which contain the anion and cation in the same molecule. These ILs were synthesized as less toxic and more sustainable variant of [*C*_2_ *mim*][*OAc*]. The authors used Martini simulations to assess the toxicity of their de novo designed ILs. It was found that the effect of the new ILs on model yeast membranes was less perturbing than for the original IL. Thus, it was concluded that the new solvents are less toxic to microorganisms. These conclusions were verified experimentally by computing the minimum inhibitory concentration.^[^ ^286^ ^]^ This study illustrates how Martini can be used in a more complete design process to also assess toxicity. However, ILs are not the only class of green solvents that can be simulated with Martini. Vainikka et al. have used Martini to simulate extraction processes with deep eutectic solvents (DES).^[^ ^290^ ^]^ This class of comparatively new molecules showing similar properties to ILs, however, have advantages in terms of cost efficiency and physical properties.^[^ ^291^ ^]^

## 4 Outlook

### 4.1 Martini 3: New Opportunities

Recent identification of some of the limits of the current Martini version,^[^ ^6^, ^27^ ^]^ opened the way for the development of a new version, coined Martini 3.^[^ ^292^ ^]^ This new version's more general re‐parametrization strategy, which did not exclusively include biomolecules, is expected to further boost the application of Martini in soft materials science. Areas in materials science that are particularly expected to benefit from the new re‐parametrization are applications involving: polymers, which constitute the backbone of soft materials science given their high tunability; conjugated molecules, which are ubiquitous in materials given the possibility of exploiting them as self‐assembling systems with interesting (opto)electronic properties; and charged systems, which are important for applications ranging from ionic liquids for green solvents to polyelectrolytes for exchange membranes and next‐generation energy storage devices. Moreover, the Martini 3 parametrization has taken into account not only infinite dilution properties such as free energies of transfer but also miscibility data on binary mixtures.^[^ ^292^ ^]^ As a consequence, the re‐calibrated Martini interaction matrix is expected to perform better in applications involving relative miscibility, self‐assembly, and aggregation propensities. Additionally, molecular packing is more accurate, as demonstrated for example in a recent biomolecular study where Martini 3 small molecules were able to find and bind to protein pockets in a wide range of systems with very high accuracy.^[^ ^293^ ^]^ Such results are promising also in view of materials studies were molecular packing is critical: moreover, the improved molecular packing implies that stacking distances between aromatic systems, which were off due to the size of Martini small beads,^[^ ^53^ ^]^ are more accurate in Martini 3. Overall, we anticipate the new version of the model to show improved predictions of molecular packing and interactions in general.

In Martini 2, the need for model refinement sometimes led to the development of custom beads (e.g., see refs. [29, 30, 68, 223]). This need often emerged when parametrizing polymers, where a slight mismatch in the properties of a monomer can build up into relatively large deviations of the macromolecular properties, and in some cases due to a suboptimal parametrization of the smaller bead sizes of the Martini model.^[^ ^27^ ^]^ However, the development of custom beads is a time‐consuming process and can limit the applicability of a Martini model if the bead is not validated properly. The upcoming new version of Martini 3 includes more generic interaction modifiers, generally dubbed “ labels”. Besides the hydrogen‐bonding labels already present in Martini 2, which have been expanded and can now^[^ ^292^ ^]^ be applied to all the new N‐ and P‐bead types, also electron polarizability labels, which mimic the electron‐donor or electron‐acceptor character of certain aromatic fragments, and self‐interaction labels, which more generically decrease/increase the self‐interaction of a certain bead type without changing its free energy of transfer, were introduced.^[^ ^292^ ^]^ Such labels expand the capabilities of Martini by giving the user a wider selection of pre‐calibrated bead types. Martini users can now use such extra bead types to fine‐tune a certain model. Accordingly, we expect the introduction of the more generic interaction modifiers in Martini 3 to greatly reduce the need for custom beads and allow for quick model refinement.

With the improved balance of interactions and possibility of model refinements, we expect Martini 3 to be suited to the description of an even wider range of systems. For example, efforts ongoing in our group are tackling the description of polyelectrolyte complex coacervates, which have material applications in adhesives, coatings, and pharmaceutical applications, aedamers, aromatic molecules that mimic biomolecules as they self‐assemble and fold into ordered states, or metal–organic frameworks (MOFs), which are of extreme interest for applications such as hydrogen storage or high‐capacity adsorbents for various separation necessities. Although Martini 3 shows numerous improvements, limitations inherent to this CGing approach remain. Of general relevance to both material and biomolecular applications is the limited structural detail due to the CGing process itself. For applications requiring very fine descriptions, atomistic or structure‐based CG approaches are more suitable.^[^ ^294^, ^295^ ^]^ Another limitation of general relevance is the entropy–enthalpy compensation. As the entropy of the system necessarily reduces due to the loss of internal degrees of freedom upon CGing, it is compensated by enthalpy to reproduce free energies. Such entropy–enthalpy imbalance is, for example, known to affect the temperature dependence of several properties, and should therefore be kept in mind. More specifically to materials systems, the description of bare metals, such as the one that may be needed to describe a metallic surface, has not been part of the parametrization and, although not impossible, requires careful validation of the chosen bead types.

### 4.2 High‐Throughput Materials Design

High‐throughput screening of soft matter is an area of immense promise for materials science.^[^ ^296^ ^]^ Ideally, a small subset of soft materials would be obtained out of a computational screening procedure so as to speed up and lower the cost of the experimental step. Given the versatility and compatibility of Martini and the efficiency gain with respect to atomistic simulations, Martini simulations are in the position to contribute to the computational design of soft materials. Some applications we envision include: 1) Design of molecular dopants with tailored miscibility: molecular doping is an important strategy used to tune organic semiconductor properties.^[^ ^297^ ^]^ There are many factors that affect the efficiency of molecular dopants, one of which is miscibility with the host semiconductor.^[^ ^297^ ^]^ Martini simulations can be used to screen molecular dopants of different polarities for insights in their miscibility with a given host semiconductor. Pushing forward studies such as refs. ^185^, ^196^ ^]^, which investigated the miscibility of only few molecular dopants, many dopant designs could be inexpensively explored with Martini and miscibility design rules extracted from such simulations. Such or similar efforts will have to be coupled with a parallel screening of said dopants’ electronic properties, which could be obtained by quantum chemical methods. 2) Design of green solvents: The proof‐of‐concept showcases of benzene and omega‐3 fatty acid extractions with Martini 3^[^ ^285^ ^]^ show promise for the usefulness of Martini in the computational design of green solvents for selective extraction using ionic liquids. Along the lines of Huet and co‐workers,^[^ ^286^ ^]^ moreover, Martini can be used to design green, biocompatible solvents that are less toxic and more sustainable. Different co‐solvents, structural motifs of the ionic liquid, or mixtures could be computationally screened in order to optimize extraction of certain molecules or other properties of the solvent such as the predicted toxicity. Again, we anticipate the recalibrated Martini 3 interaction matrix, validated by taking into account miscibility data, to be a great asset for such studies. 3) Determining molecular structure–morphology relationships in self‐assembling materials: In order to rationally design self‐assembling materials for specific applications, one needs to derive robust molecular structure–morphology relationships of the final aggregate or melt. However, the chemical space of organic materials is extremely vast, even if one puts some molecular constraints dictated by the specific application. Many molecular features are known to affect the final morphology of soft materials, but extracting robust rules is very time and resource intensive with experiments alone. Given the compatibility of Martini models and the ease with which one can vary features such as polarity of molecular features, side‐chain lengths, and molecular topology, we anticipate that Martini simulations aimed at exploring such parameter space in a high‐throughput fashion are possible. These efforts, especially if combined with experimental feedback loops and validation, will help reach the overarching goal of establishing robust structure–morphology relationships in materials systems ranging from self‐assembling supramolecular materials, over thin films for organic electronics, to nanoparticle–polymer composites.

The above envisioned applications necessarily reflect the own biases of the authors, and of course many more applications can be conceived along these lines and beyond.

To fully harness the compatibility of Martini models and the growing access to computational power, and hence realize high‐throughput workflows, the development of tools to automate the Martini workflow will be of key importance. Such tools need to include programs able to automatically create Martini models or Martini building blocks for large molecules. This means they need to design the mapping, assign bead types, and derive bonded interactions from atomistic simulations. Furthermore, tools to generate force field files from such building blocks are required as well as tools to create initial coordinates for a variety of target systems. Such tools would not only accelerate the making of Martini models needed for truly high‐throughput pipelines, but also make the process more robust and reliable. Endeavors in this direction are already ongoing with tools such as AutoMARTINI^[^ ^298^ ^]^ or Cartographer,^[^ ^299^ ^]^ which are able to derive atomistic‐to‐Martini mappings, as well as perform a basic bead‐type assignment. While the proof of concepts are promising more robust ways for assigning bead types and mappings will need to be found. This is especially true for Martini 3, which includes more bead types, bead sizes, and refined mapping rules. For parametrization of CG bonded interactions from atomistic simulations PyCGTOOL^[^ ^300^ ^]^ and Swarm‐CG^[^ ^301^ ^]^ have been designed. Whereas PyCGTOOL is able to efficiently derive bonded interactions for small molecules, it is often impractical for large molecules such as polymers. For example, it cannot recognize and optimize redundant bonded interactions. This deficit is overcome by Swarm‐CG, which uses a machine‐learning‐based optimization approach. However, currently generating interactions for medium‐sized molecules is still comparatively slow and not all bonded interaction types are implemented. This includes some of those especially important in materials science such as the restricted bending potential or the combined bending and torsion potential.^[^ ^33^ ^]^ The Swarm‐CG tool, at the time of writing, is still actively being optimized on this aspect. Recently, the development of Martinize 2^[^ ^302^ ^]^ aims at mapping an entire system from an atomistic reference structure to Martini, generating both target coordinates and input files based on already parametrized fragments. This will allow more rigorously transforming all‐atom systems to Martini resolution in one go without having to rebuild the system from the individual components. Together with backward,^[^ ^191^ ^]^ this will also allow resolution transformation in both directions introducing atomistic detail when needed but also capturing that detail when transforming back to Martini.

**Figure 10:**
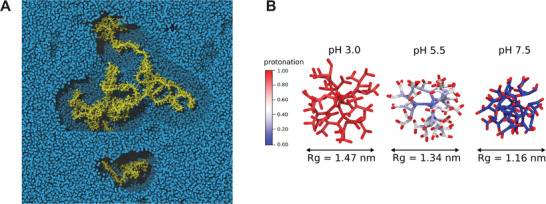
Modeling of materials with Martini: recent developments. A) Melt of Martini *PS*_1000_ generated using Polyply.^[^ ^65^ ^]^ Highlighted in gold is a single chain with other chains (blue spheres) removed within a cut‐off of 2.3 nm. The total system size is about (30 nm)^3^. B) Protonation of a poly(propylene imine) (PPI) dendrimer as a function of pH as modeled with the titratable version of Martini.^[^ ^303^ ^]^ The protonation state of the core beads of the dendrimer, which represent tertiary amines, clearly changes as a function of pH, becoming progressively less protonated as indicated with the color scale from red (protonated most of the time) to blue (deprotonated most of the time). The radius of gyration (Rg) quantifies the degree of polymer collapse as the charge density decreases at higher pH. B) Reproduced with permission.^[^ ^303^ ^]^ Copyright 2020, American Institute of Physics.

Besides model parametrization and resolution transformation, tools to setup starting structures will also be of increasing importance as the complexity of the simulated systems grows. In this context, recently, Grünewald and co‐workers developed Polyply, a software suite for facilitating atomistic and CG polymer simulations. The tool can generate topologies of polymer systems ranging from simple or complex homopolymers, over branched and hyperbranched polymers, to block copolymers.^[^ ^65^ ^]^ Not only single‐chain starting structures but also melts and more complex pre‐assembled morphologies can be generated. The latter strategy is useful, because whereas self‐assembly might be the preferred strategy to assemble a polymer morphology, it remains a challenging task even at the Martini CG level due to the slow dynamics of long polymer chains. For example, **Figure** **10**A shows a PS melt system with a molecular weight of 1000 residues. Highlighted in gold is a single chain, which winds and twists almost from one edge to the other. The total dimensions of the system is about (30 nm)^3^ and comprises half a million CG particles. While properly mixing such a melt from initially disentangled chains is an almost impossible undertaking, Polyply generates such structures within minutes. Polyply's internal library already contains several Martini polymer models from the literature but more models can be contributed via GitHub.^[^ ^65^ ^]^ This tool is expected to standardize and greatly simplify the generation and setup of Martini polymer systems.

Other tools with a similar purpose are also being developed. For example, CHARMM‐GUI already supports generation of Martini membranes^[^ ^304^ ^]^ and sugars,^[^ ^305^ ^]^ and is currently being extended to polymers. Whereas Polyply and CHARMM‐GUI can generate both topologies and structures, PACKMOL^[^ ^306^ ^]^ is a tool that can be used to generate starting structures from existing molecule coordinates.

Maintenance of a library of available Martini models, and their curation, will also be important on this front. Many Martini models are available at http://cgmartini.nl: besides an extensive number of biomolecules, and in particular lipids, a growing “polymerdome,” and upcoming extensive Martini 3 solvent^[^ ^292^ ^]^ and small‐molecule^[^ ^307^ ^]^ databases will be important for materials science applications. Moreover, both the upcoming Martinize 2^[^ ^302^ ^]^ and Polyply^[^ ^65^, ^66^ ^]^ tools rely on the Vermouth library,^[^ ^302^ ^]^ which offers a consistent way of defining building blocks and keeping track of model versions, thereby contributing to better data curation which is an important aspect of growing data sets. For example, researchers can contribute their polymer models to the Polyply library on the GitHub page,^[^ ^65^ ^]^ which implements a quality control procedure ensuring correctness of produced topology files. Models, contributed in this way, can be used to generate polymer sequences of custom length and composition fitting to the researchers specific problems without the need for manually curating models.

### 4.3 Advanced Martini Simulations

The growing computational power available and the increase in the complexity of the simulated systems demands for smart and (semi‐)automated ways to drive, explore, and analyze the simulated system. Moreover, combining Martini with new method developments can open the way for advanced simulations that go beyond what is feasible with standard CG MD.

Despite the growing computational power available, brute‐force sampling of the conformational space often is still not sufficient. Enhanced sampling techniques are available to increase the effective simulation time of MD simulations in general. A host of enhanced sampling techniques already exist and it is in continuous expansion, and many techniques are implemented in packages such as PLUMED^[^ ^308^ ^]^ and SSAGES,^[^ ^309^ ^]^ which are compatible with many MD softwares, including GROMACS^[^ ^34^ ^]^ and NAMD,^[^ ^310^ ^]^ and hence can be readily applied to Martini systems. Given the insights such techniques have already provided when applied to Martini simulations^[^ ^258^, ^264^ ^]^ and the active developments in this area, Martini simulations will surely benefit from coupling to such techniques.

Changes in bonded interactions and atom types, which reflect chemical reactions, cannot be captured by regular MD simulations. However, chemical reactions especially in the field of material science are ubiquitous. Examples include cross‐linking reactions in polymers, dynamic changes in the protonation states of polyelectrolytes, or formation of gels. Efforts are being taken toward capturing these effects.^[^ ^89^, ^98^, ^303^, ^311^ ^]^ Rossi et al., for example, studied the cross‐linking of a polyester resin. To model a cross‐linking reaction they used an ad hoc empirical potential, which displayed harmonic‐like features at close distance, but was able to dissociate at larger distance. As another example, Zadok and Srebnik used a LAMMPS built‐in feature to simulate the effects of gel formation for a protein‐imprinted hydrogel.^[^ ^89^ ^]^ Ghermezcheshme and co‐workers used a similar approach, however, combining GROMACS with an in‐house code to study the step‐growth reaction of polyurethane. Here, a bond is introduced after finding all reactive neighbors within a cut‐off. Upon changing the topology a long equilibrium simulation is carried out after which another step of cross‐linking is performed. In contrast, the recently developed titratable Martini^[^ ^303^ ^]^ allows the dynamic representation of protonation reactions in a Martini simulation. Here the protonation state of a titratable functional group can change back and forth between protonated and deprotonated during the course of a continuous simulation. Using this approach, for example, the pH‐induced collapse of the hyperbranched polymer poly(propylene imine) was simulated as shown in Figure 10B. Like the approach by Rossi, an empirical nonbonded potential is used to allow protons to tightly bind to titratable functional groups. Further progress in the field of reaction simulations is expected with the inclusion of lambda dynamics into GROMACS.^[^ ^34^ ^]^

Machine learning (ML) approaches coupled to molecular modeling are emerging in soft materials research.^[^ ^312^, ^313^ ^]^ Such approaches have the potential to support and augment traditional physics‐based models in computational research.^[^ ^314^ ^]^ In the realm of soft materials, ML techniques have been shown to be able to predict electronic properties, such as energy levels and absorption spectra of soft materials directly from CG structures.^[^ ^315^, ^316^ ^]^ This strategy is a potentially quicker and less laborious alternative to the currently necessary backmapping and subsequent quantum chemical calculation step, and it is especially relevant to systems where Martini structures are usually backmapped to obtain electronic properties, such as organic electronic systems.^[^ ^192^, ^193^, ^194^ ^]^ Another interesting avenue is the one of coupling CG simulations and ML techniques to efficiently explore the desired chemical space. For example, Shmilovic and co‐workers^[^ ^256^ ^]^ used Martini simulations within an active learning strategy to efficiently cover the chemical space span by π‐conjugated peptides with a π‐core flanked by two tripeptide units. Instead of simulating all possible tripeptide sequences (20^3^ = 8000), the active learning strategy allowed the amount of simulation data needed for training the ML model to be minimized. Accordingly, by direct simulations of only 2.3% of the tripeptide space, the authors showed how a Gaussian process regression model could capture the statistical information necessary to represent this chemical space.^[^ ^256^ ^]^ The model could then be leveraged to identify the π‐conjugated peptides predicted to exhibit superior assembly properties to those reported in previous work. Moreover, in this way, the authors were able to reveal design rules governing assembly of these molecules. The fact that the Martini bead types discretize the chemical space, means that similar molecules will often map to the same Martini CG model. This introduces a degeneracy in the CG representation, which translates into a reduction of the size of the chemical (compound) space.^[^ ^317^ ^]^ Accordingly, this reduction of the size of the chemical space represents also a further speed‐up which can help for screening studies.^[^ ^317^ ^]^ Hence, we expect hybrid Martini/machine‐learning schemes to be highly promising in order to efficiently explore the chemical space for different applications.

In conclusion, we foresee a bright perspective for applications of the Martini model in the field of materials science, especially given the possibilities offered by the new version of the force field, existing and forthcoming tools to streamline model building and system preparation, and combination with existing and future method developments, such as constant pH simulations and hybrid simulation/machine learning schemes.

## Conflict of Interest

The authors declare no conflict of interest.
